# Deletion of the *GAPDH* gene contributes to genome stability in *Saccharomyces cerevisiae*

**DOI:** 10.1038/s41598-020-78302-5

**Published:** 2020-12-03

**Authors:** Miki Hanasaki, Keisuke Yaku, Motohiro Yamauchi, Takashi Nakagawa, Hiroshi Masumoto

**Affiliations:** 1 Biomedical Research Support Center (BRSC), Nagasaki University School of Medicine, 1-12-4 Sakamoto, Nagasaki, Nagasaki 852-8523 Japan; 2 Department of Metabolism and Nutrition, Graduate School of Medicine and Pharmaceutical Sciences for Research, University of Toyama, 2630 Sugitani, Toyama, Toyama 930-0194 Japan; 3 Department of Radiation Biology and Protection, Atomic Bomb Disease Institute, Nagasaki University, 1-12-4 Sakamoto, Nagasaki, Nagasaki 852-8523 Japan

**Keywords:** Genomic instability, DNA metabolism

## Abstract

Cellular metabolism is directly or indirectly associated with various cellular processes by producing a variety of metabolites. Metabolic alterations may cause adverse effects on cell viability. However, some alterations potentiate the rescue of the malfunction of the cell system. Here, we found that the alteration of glucose metabolism suppressed genome instability caused by the impairment of chromatin structure. Deletion of the *TDH2* gene, which encodes glyceraldehyde 3-phospho dehydrogenase and is essential for glycolysis/gluconeogenesis, partially suppressed DNA damage sensitivity due to chromatin structure, which was persistently acetylated histone H3 on lysine 56 in cells with deletions of both *HST3* and *HST4,* encoding NAD^+^-dependent deacetylases. *tdh2* deletion also restored the short replicative lifespan of cells with deletion of *sir2*, another NAD^+^-dependent deacetylase, by suppressing intrachromosomal recombination in rDNA repeats increased by the unacetylated histone H4 on lysine 16. *tdh2* deletion also suppressed recombination between direct repeats in *hst3*∆ *hst4*∆ cells by suppressing the replication fork instability that leads to both DNA deletions among repeats. We focused on quinolinic acid (QUIN), a metabolic intermediate in the de novo nicotinamide adenine dinucleotide (NAD^+^) synthesis pathway, which accumulated in the *tdh2* deletion cells and was a candidate metabolite to suppress DNA replication fork instability. Deletion of *QPT1*, quinolinate phosphoribosyl transferase, elevated intracellular QUIN levels and partially suppressed the DNA damage sensitivity of *hst3*∆ *hst4*∆ cells as well as *tdh2*∆ cells. *qpt1* deletion restored the short replicative lifespan of *sir2*∆ cells by suppressing intrachromosomal recombination among rDNA repeats. In addition, *qpt1* deletion could suppress replication fork slippage between direct repeats. These findings suggest a connection between glucose metabolism and genomic stability.

## Introduction

Genome instability is closely connected with both carcinogenesis and aging^1–3^. DNA damage is an alteration in the chemical structure of DNA. Common types of DNA damage include DNA base modifications, DNA inter- and intrastrand crosslinks, and DNA single- and double-strand breaks (SSBs and DSBs, respectively)^4^. Endogenous and exogenous sources of DNA damage lead to genomic instability (reviewed in^5^). Endogenous sources of DNA damage include reactive oxygen species (ROS) or some other products of DNA metabolism. Endogenous sources of DNA damage can lead to DNA base modifications and the formation of bulky adducts. Problems in DNA metabolism (e.g., DNA replication and chromosomal segregation) can lead to DNA breaks (SSBs and DSBs). Exogenous sources of DNA damage are external agents, including ionizing radiation, ultraviolet radiation and a variety of chemical agents. Chemical agents can have various effects on the DNA strand (e.g., DNA intercalation, DNA crosslinking, and DNA alkylation). *Cis*-elements of the DNA sequence affect replication fork stability. The replication fork can skip among DNA repeats (e.g., trinucleotide repeats, inverted repeats or direct repeats), resulting in deletions or mutations^1,6–9^. The proteins involved in DNA damage repair, DNA replication and the cell cycle checkpoint work cooperatively to maintain genome integrity to fix DNA lesions or to prevent DNA replication fork instability. Mutations of these proteins drastically cause the accumulation of mutations in the chromosome, which results in carcinogenesis and progeria^2,3,9^.

Chromatin is composed of DNA fiber and chromatin-binding proteins such as histones that package chromosomal DNA into nuclei. The condensed structure usually becomes an obstacle for the execution of nuclear activities on chromatin. Therefore, chromatin regulator proteins (e.g., histone modifiers and chromatin remodeling factors) create an environment that allows the replisome, transcriptional machinery and DNA damage repair machinery to perform on chromatin^10,11^. The canonical histones (H2A, H2B, H3 and H4) harbor various posttranslational modifications (such as acetylation, methylation, phosphorylation and ubiquitination)^10^. Histone acetyltransferase (HAT) and histone deacetylase (HDAC) acetylate and deacetylate lysine residues in histone proteins, respectively. HAT and HDAC cooperatively manage gene transcription^12,13^, chromatin remodeling^14^, and DNA damage repair^15,16^. Hst3 and Hst4, NAD^+^-dependent deacetylases in budding yeast^17^, mutually deacetylate histone H3 on lysine 56 (H3-K56)^18–20^. Histone H3-K56 is persistently acetylated in *hst3*∆ *hst4*∆ cells, which causes sensitivity to DNA damaging agents because of the loosened chromatin structure^18,19^. Because the deletion of the *RTT109* gene, encoding the HAT responsible for histone H3-K56 acetylation, allows histone H3-K56 to be deacetylated and confers severe DNA damage sensitivity, proper control of both acetylation and deacetylation on histone H3-K56 in chromatin is needed to perform DNA repair^21^. Sir2 is another NAD^+^-dependent deacetylase, and one substrate of Sir2 is histone H4 on K16^22^. Sir2 is involved in gene silencing of genes inserted within ribosomal DNA (rDNA) repeats and at telomere loci and the silent mating type loci *HML* and *HMR*^23^. *sir2* deletion causes persistence of histone H4 acetylation on K16 in chromatin, which elevates the frequencies of intrachromosomal recombination in rDNA repeats by replication fork slippage and generates extrachromosomal ribosomal DNA circles (ERCs)^24,25^. The accumulation of ERCs reduces the replicative lifespan in *sir2*∆ cells^24,25^. Thus, persistence of either H3-K56 acetylation on chromatin or H4 K16 acetylation on rDNA repeats leads to genome instability.

In this study, we found that the alteration of glucose metabolism suppressed the genome instability caused by aberrant chromatin structure. Deletion of the *TDH2* gene, which encodes glyceraldehyde 3-phosphate dehydrogenase (GAPDH), a metabolic enzyme in glycolysis/gluconeogenesis, partially suppressed the DNA damage sensitivity of *hst3*∆ *hst4*∆, although the *tdh2* gene deletion did not affect the H3-K56 acetylation in *hst3*∆ *hst4*∆ cells. *tdh2* deletion could also restore the short replicative lifespan of *sir2*∆ cells. In addition, *tdh2* deletion could suppress replication fork slippage between direct repeats in both wild-type and *hst3*∆ *hst4*∆ cells. Furthermore, we examined the role of quinolinic acid (QUIN), which is a metabolic intermediate in the de novo nicotinamide adenine dinucleotide (NAD^+^) synthesis pathway (or kynurenine pathway) and was accumulated in *tdh2∆* cells, and tested whether QUIN could suppress replication fork instability as observed in *tdh2*∆ cells. The cells with deletion of *QPT1*, quinolinate phosphoribosyl transferase, which synthesizes nicotinate mononucleotide (NaMN) from QUIN, experienced elevated intracellular QUIN concentrations and partially suppressed DNA damage sensitivity in *hst3*∆ *hst4*∆ cells similarly as observed in *tdh2∆* cells. The *qpt1* deletion restored the short replicative lifespan of *sir2*∆ cells. Furthermore, the *qpt1* deletion suppressed replication fork slippage between direct repeats more compared to wild-type cells. These findings suggest that metabolic alteration contributes to preventing the genomic instability caused by the impaired function of chromatin regulation, and QUIN may be a candidate to stabilize the DNA replication fork to prevent DNA damage.

## Results

### Deletion of the *TDH2* gene partially suppresses the DNA damage sensitivity of *hst3*∆ *hst4*∆ cells

**Figure 1:**
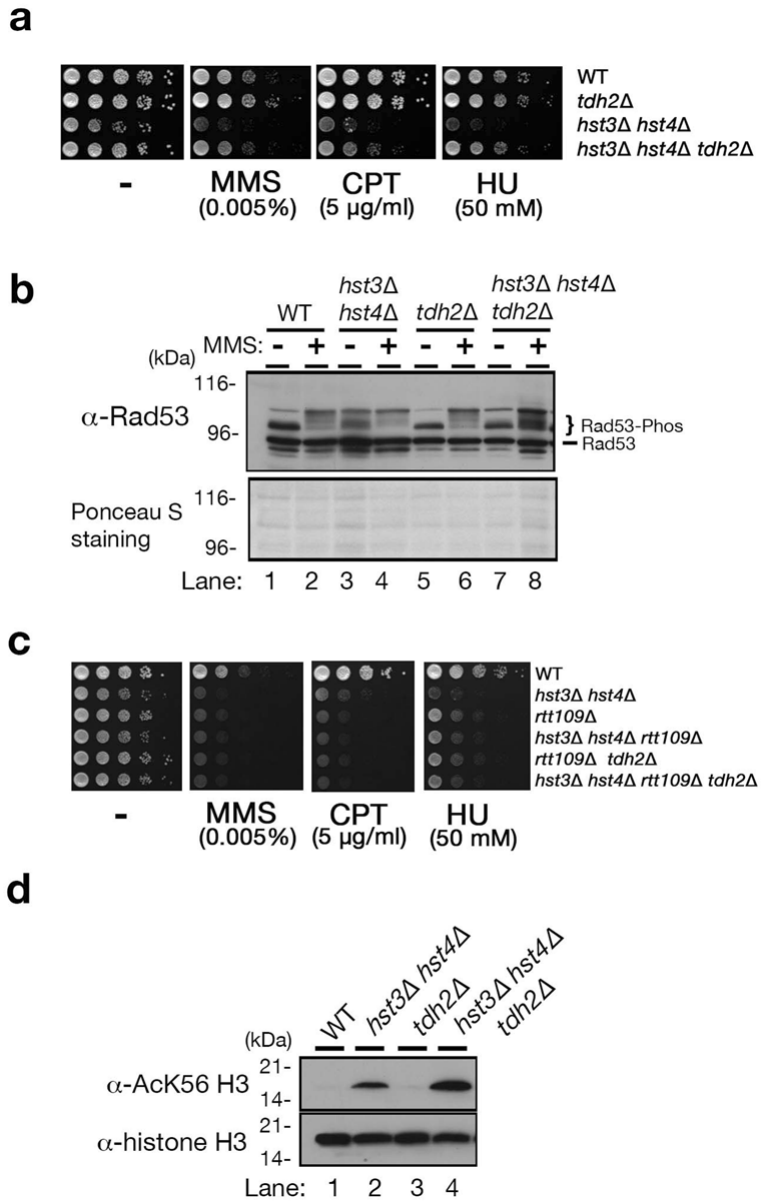
*tdh2* gene deletion can suppress the DNA damage sensitivity of *hst3*∆ *hst4*∆ cells. (**a**,**c**) Plate assays monitoring the sensitivity of each DNA damaging agent (methyl methanesulfonate (MMS), hydroxyurea (HU) and camptothecin (CPT)). A ten-fold dilution of the cell suspension was sequentially diluted (from left to right) and then spotted on solid medium. WT: wild-type. (**b**) Rad53 phosphorylation was detected by western blotting using an anti-Rad53 antibody. Ponceau S staining indicates the total amount of protein on the membrane. (**d**) Acetylation level of histone H3-K56 on G2/M phase-arrested cells.

The *TDH2* gene encodes one of the yeast GAPDH genes (*TDH1/2/3*) involved in both glycolysis and gluconeogenesis. A previous study reported that *hst3*∆ *hst4*∆ cells activate gluconeogenesis in the presence of glucose, and the *tdh2* gene deletion represses gluconeogenesis in enhanced *hst3*∆ *hst4*∆ cells and restores slow growth^26^. Because the slow growth of *hst3*∆ *hst4*∆ cells is due to the frequent occurrence of DNA damage during cell cycle progression^18,19^, we tested whether the *tdh2* gene deletion was able to suppress the DNA damage sensitivity of *hst3*∆ *hst4*∆ cells. This was examined by monitoring cell growth in YPD (Yeast extract-Polypeptone-Dextrose) solid medium supplemented with the following DNA damaging agents: methyl methanesulfonate (MMS), hydroxyurea (HU) and camptothecin (CPT). The alkylating agent MMS attaches alkyl groups to DNA bases, and HU inhibits ribonucleotide reductase to reduce intracellular deoxyribonucleotide levels. CPT inhibits Type I topoisomerases, thus DNA SSBs are not repaired, and then creates a DNA DSB after passing the replication fork. These agents promote replication collapse and eventually cause DNA DSBs, which are usually fixed by homologous recombination (HR)^8,16,27^. *hst3*∆ *hst4*∆ *tdh2*∆ cells grew in YPD solid medium amended with each DNA damaging agent better than *hst3*∆ *hst4*∆ cells; however, the growth was not restored to the levels of the wild-type and *tdh2*∆ cells (Fig. 1a and Fig. S1). Rad53 is a member of both the DNA damage and the intra-S phase checkpoint systems and harbors multiple phosphorylation sites phosphorylated by checkpoint activation in response to DNA lesions or replication fork arrests^28–34^. The extent of Rad53 phosphorylation depends on the intensity of DNA damage. We monitored Rad53 phosphorylation to examine whether deleting *tdh2* represses the occurrence of DNA damage in *hst3*∆ *hst4*∆ cells. Multiple phosphorylated Rad53 was indicated in wild-type and *tdh2*∆ cells treated with MMS by the presence of slow-migrated bands in sodium dodecyl sulfate–polyacrylamide gel electrophoresis (SDS-PAGE), although a single band of unphosphorylated Rad53 was detected in cells without MMS (Fig. 1b; lanes 1, 2, 5 and 6). Phosphorylated Rad53 bands were detected in *hst3*∆ *hst4*∆ and *hst3*∆ *hst4*∆ *tdh2*∆ cells treated with MMS, similar to the patterns of wild-type and *tdh2*∆ cells (Fig. 1b; lanes 2, 4, 6 and 8). Even in the absence of MMS, a smeared Rad53 band was detected in *hst3*∆ *hst4*∆ cells (Fig. 1b; lane 3), indicating that the chromatin acetylated at histone H3-K56 becomes fragile and induces DNA damage during nuclear activities^18,19^. Interestingly, the smeared Rad53 band was not detected in *hst3*∆ *hst4*∆ *tdh2*∆ cells without MMS treatment or in wild-type cells (Fig. 1b; lanes 1 and 7). Thus, *tdh2* deletion suppresses the occurrence of DNA damage originating from the chromatin associated with persistently acetylated histone H3-K56 in *hst3*∆ *hst4*∆ cells. Next, we asked whether DNA damage suppression by *tdh2* deletion was restricted to the chromatin acetylated histone H3-K56. Rtt109 functions as an acetyltransferase for histone H3-K56^21^. *rtt109*∆ cells, which did not acetylate histone H3-K56, showed greater sensitivity to MMS, CPT and HU than *hst3*∆ *hst4*∆ cells^21^ (Fig. 1c and Fig. S2). The DNA damage sensitivity of *hst3*∆ *hst4*∆ *rtt109*∆ cells was the same as that of *rtt109*∆ cells, in that H3-K56 in chromatin remained deacetylated in *hst3*∆ *hst4*∆ *rtt109*∆ (Fig. 1c and Fig. S2). Both *rtt109*∆ *tdh2*∆ and *hst3*∆ *hst4*∆ *rtt109*∆ *tdh2*∆ cells exhibited the same DNA damage sensitivities as *rtt109*∆ cells (Fig. 1c and Fig. S2), suggesting that *tdh2* deletion does not suppress the occurrence of DNA damage caused by chromatin deacetylated histone H3-K56. Next, we asked whether *tdh2* deletion might reduce the acetylation level of histone H3-K56 in *hst3*∆ *hst4*∆ cells and gain resistance to DNA damage sensitivity. Our previous analysis showed that the level of acetyl-CoA, which is used as a substrate to acetylate lysine residue on target proteins, including histones, was increased in *hst3∆ hst4∆ tdh2∆* cells more than in wild-type, *hst3∆ hst4∆* and *tdh2∆* cells^26^. To exclude the possibility that another histone modifier reduced the acetylation level of histone H3-K56 to a level sufficient to promote DNA damage repair in *hst3*∆ *hst4*∆ *tdh2*∆ cells, we examined whether *tdh2* deletion influenced the H3-K56 acetylation level in *hst3*∆ *hst4*∆ cells. The acetylation of histone H3-K56 is regulated in a cell cycle-dependent manner, and this acetylation appears from late G1 to early G2 phase^16,35^. When the G2/M phase was arrested by treatment with nocodazole, an inhibitor of microtubule polymerization, H3-K56 was deacetylated in both wild-type and *tdh2*∆ cells (Fig. 1d; lanes 1 and 3). In contrast, the acetylation level of histone H3-K56 remained the same between *hst3*∆ *hst4*∆ and *hst3*∆ *hst4*∆ *tdh2*∆ cells treated with nocodazole (Fig. 1d; lanes 2 and 4). Thus, *tdh2* deletion does not affect the acetylation level in *hst3*∆ *hst4*∆ cells. Altogether, *tdh2* deletion is able to suppress the genome instability caused by the aberrant chromatin structure constitutively acetylated histone H3 on K56.

### *tdh2* deletion suppresses deletion between direct repeats (DRs) on the chromosome

**Figure 2:**
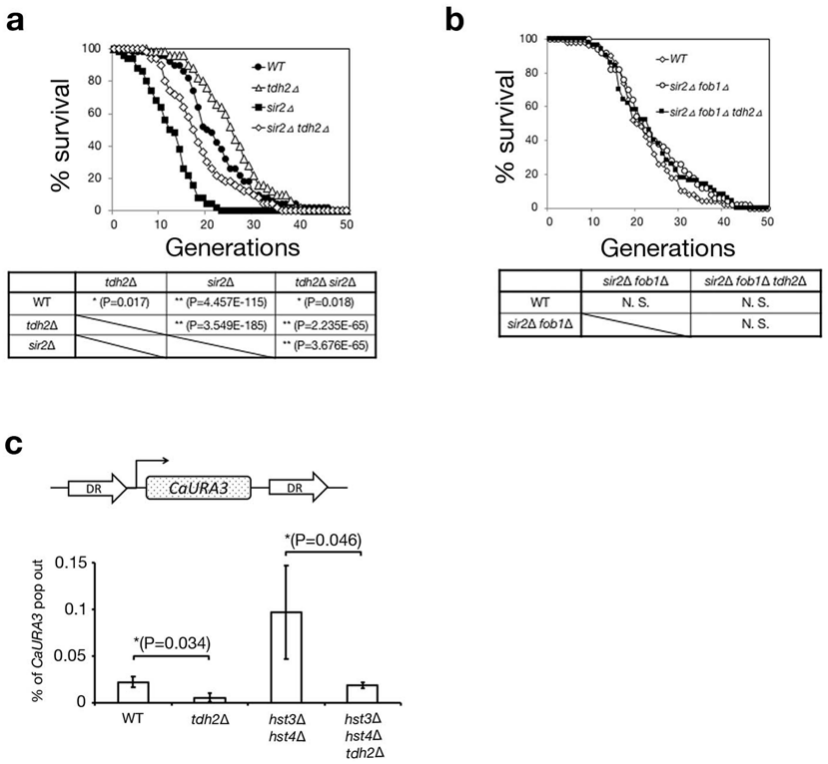
*tdh2* gene deletion can suppress intrachromosomal recombination among repeats. (**a**,**b**) Pedigree analysis to count replicative lifespans among cells. *P < 0.05. **P < 0.01. N.S: not significant. Unpaired t-test (two-tailed). Over 50 cells/strain were used for analysis. (**c**) The frequencies of CaURA3 gene deletion among repeats. DRs: direct repeats. *P < 0.05. **P < 0.01. N.S: not significant. Unpaired t-test (two-tails). Error bars represent the standard deviation of three biological replicates.

Next, we tested whether *tdh2* deletion could suppress the genome instability caused by chromatin acetylation other than H3-K56. The *sir2* deletion increases the acetylation level of histone H4 on K16 at ribosomal DNA (rDNA) repeats and elevates the ratio of intrachromosomal recombination among rDNA repeats by replication fork slippage to bear ERCs^25^. Accumulation of ERCs reduces the replicative lifespan of *sir2*∆ cells^36^. Pedigree analysis monitors the replicative age of mother cells to count the number of divided daughter cells. The replicative lifespan of wild-type cells was approximately 22 divisions (50% viability), with a maximum of approximately 40 (Fig. 2a; wild-type). The lifespan of *sir2*∆ cells was significantly reduced to half the level (approximately 13 divisions (50% viability)) of wild-type cells (Fig. 2a; wild-type *vs*. *sir2*∆ (*P* = 4.457E-115)). *tdh2*∆ cells had an increased replicative lifespan compared to wild-type cells (Fig. 2a; wild-type *vs*. *tdh2*∆ (*P* = 0.017))^26^. *sir2*∆ *tdh2*∆ cells exhibited a significantly extended lifespan compared to the *sir2*∆ cells but did not reach the lifespan of the wild-type cells (Fig. 2a; *sir2*∆ *tdh2*∆ vs. *sir2*∆ (*P* = 3.676E-65), and *sir2*∆ *tdh2*∆ *vs*. wild-type (*P* = 0.018)). Fob1 functions as a replication fork barrier in rDNA repeats, which elevates the opportunities for intrachromosomal recombination among rDNA repeats^37^. The *fob1* deletion restored the lifespan of *sir2*∆ cells such that the lifespan of *sir2*∆ *fob1*∆ cells was almost the same as that of wild-type (Fig. 2b; wild-type vs. *sir2*∆ *fob1*∆). The replicative lifespans were almost the same among wild-type, *sir2*∆ *fob1*∆ and *sir2*∆ *fob1*∆ *tdh2*∆ cells (Fig. 2b; wild-type vs. *sir2*∆ *fob1*∆ *vs*. *sir2*∆ *fob1*∆ *tdh2*∆), suggesting that *tdh2* deletion suppresses the intrachromosomal recombination among rDNA repeats in the same manner as *fob1* deletion. These data suggest that the *tdh2* gene deletion can suppress replication fork slippage, causing intrachromosomal recombination among rDNA repeats in *sir2*∆ cells. To examine whether the genomic instability suppressed by *tdh2* deletion is due to aberrant intrachromosomal recombination between direct repeats (DRs) in *hst3*∆ *hst4*∆ cells, we used a strain to monitor the ratio of the *CaURA3* gene deletion by recombination between DRs due to replication fork slippage (Fig. 2c top). The *CaURA3* deletion cells can grow in SC solid medium containing 5-floroorotic acid (5-FOA), a counterselecting agent for *URA3* gene deletion. The frequency of *CaURA3* gene deletion in *hst3*∆ *hst4*∆ *tdh2*∆ cells was significantly (*P* = 0.046) reduced to levels lower than those of *hst3*∆ *hst4*∆ cells (Fig. 2c; *hst3*∆ *hst4*∆ *vs*. *hst3*∆ *hst4*∆ *tdh2*∆). Furthermore, the frequency of *CaURA3* gene deletion in *tdh2*∆ cells was significantly (*P* = 0.034) reduced to levels lower than those of wild-type cells (Fig. 2c; wild-type vs. *tdh2*∆). Thus, *tdh2* deletion is involved in suppressing deletions among DRs due to replication fork slippage. Next, we examined whether *tdh2* deletion supported the DNA repair machinery in preventing deletion by replication fork slippage. DNA repair machinery has a pivotal role in preventing genome rearrangement in DNA replication^38^. Sgs1, a RecQ family nucleolar DNA helicase, suppresses DNA replication-associated genome rearrangement^39,40^. *sgs1*∆ cells exhibited MMS and HU sensitivity, leading to DNA damage due to DNA replication fork stalling (Fig. S3; wild-type and *sgs1*∆). Because the HU and MMS sensitivities of *tdh2∆* cells were almost the same as those of *sgs1*∆ cells (Fig. S3; *sgs1*∆ and *sgs1*∆ *tdh2*∆), the replication fork stability by *tdh2* deletion depends on the DNA repair machinery. Thus, *tdh2* deletion increases the replication fork stability together with the DNA repair machinery.

### Quinolinic acid (QUIN) is a metabolic candidate to suppress replication fork instability

**Figure 3:**
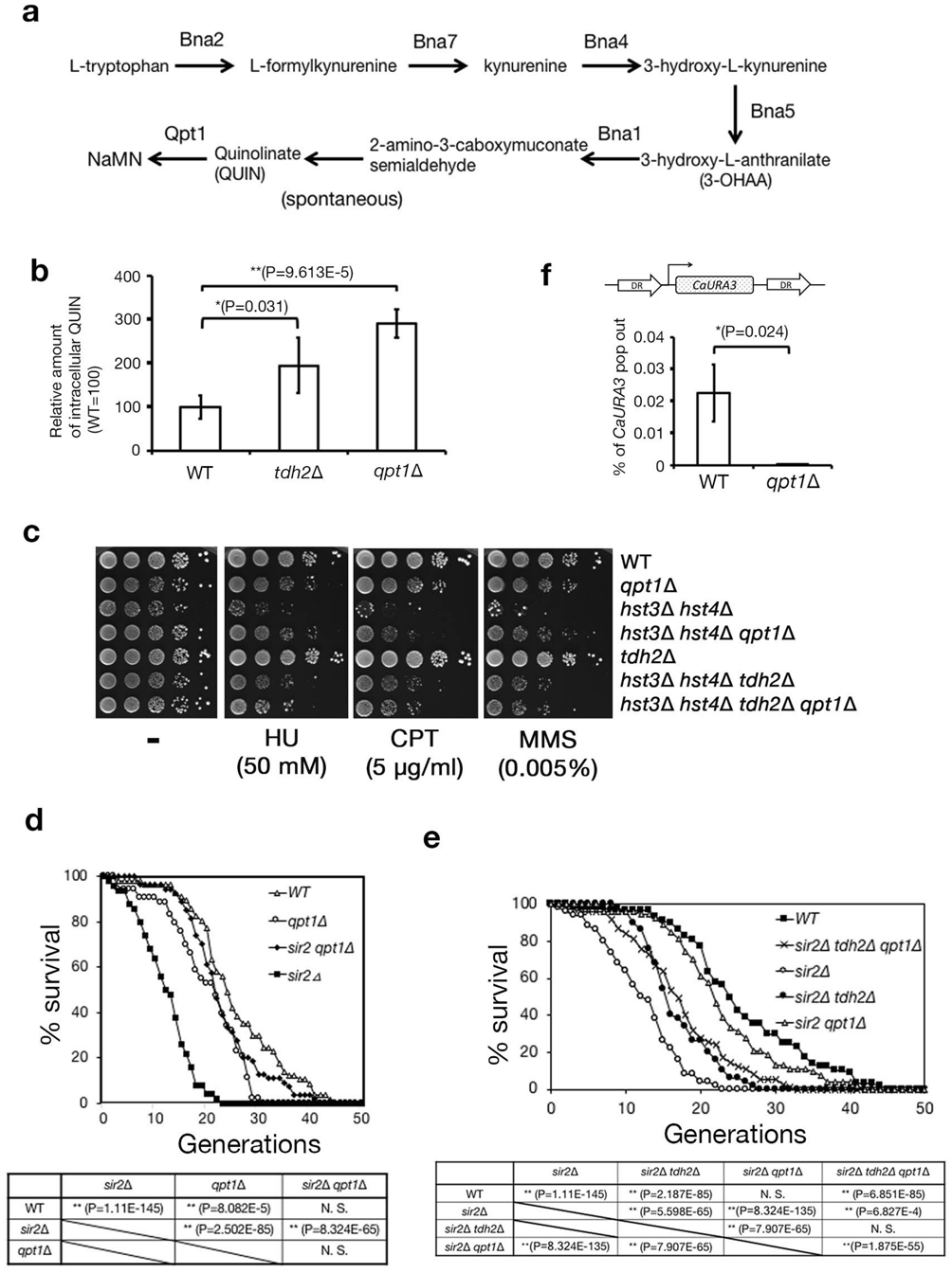
Quinolinic acid (QUIN) is a candidate metabolite that is increased in *tdh2*∆ cells to suppress replication fork slippage. (**a**) The metabolic pathway of the de novo NAD^+^ synthesis pathway (kynurenine pathway). NaMN: nicotinic acid mononucleotide. (**b**) Comparison of intracellular QUIN levels among strains. Relative amount of QUIN in each cell per wild-type cell (WT = 100). *P < 0.05. **P < 0.01. Unpaired t-test (two-tailed). Error bars represent the standard deviation of three biological replicates. (**c**) Plate assays monitoring the sensitivity of each DNA damaging agent. (**d**,**e**) Pedigree analysis to count replicative lifespans among cells. *P < 0.05. **P < 0.01. N.S: nonsignificant. Unpaired t-test (two-tails). Over 50 cells/strain were used for analysis. (**f**) The frequencies of the *CaURA3* deletion among repeats. DRs: direct repeats. *P < 0.05. Unpaired t-test (two-tailed). Error bars represent the standard deviation of three biological replicates.

We examined whether a metabolite increased in *tdh2*∆ cells might contribute to replication fork stability in both wild-type and *hst3*∆ *hst4*∆ cells. Using capillary electrophoresis time-of-flight mass spectrometry (CE-TOF/MS) analysis to compare the levels of cellular metabolites^26^, we focused on quinolinic acid (QUIN), which was significantly increased in *tdh2∆* cells. QUIN is a metabolic intermediate in the de novo NAD^+^ synthetic pathway, or the kynurenine pathway (Fig. 3a). QPT1 encodes quinolinate phosphoribosyltransferase to converts QUIN to nicotinic acid mononucleotide (NaMN). We confirmed that the amount of intracellular QUIN significantly (*P* < 0.05) accumulated in both *tdh2*∆ and *qpt1∆* cells more than wild-type cells (Fig. 3b; wild-type, *tdh2*∆ and *qpt1*∆). We used the *qpt1* deletion strain to examine whether QUIN was able to suppress the DNA damage sensitivity of *hst3*∆ *hst4*∆ cells. *hst3*∆ *hst4*∆ *qpt1*∆ cells exhibited better tolerance to DNA damaging agents (MMS, HU and CPT) than *hst3*∆ *hst4*∆ cells. However, the tolerance was not recovered to the levels of the wild-type and *tdh2*∆ cells (Fig. 3c and Fig. S4; wild-type, *qpt1*∆, *hst3*∆ *hst4*∆ *qpt1*∆ and *hst3*∆ *hst4*∆). Furthermore, the sensitivity of *hst3*∆ *hst4*∆ *tdh2*∆ *qpt1*∆ cells was almost the same as that of *hst3*∆ *hst4*∆ *tdh2*∆ cells (Fig. 3c and Fig. S4; *hst3*∆ *hst4*∆ *tdh2*∆ and *hst3*∆ *hst4*∆ *tdh2*∆ *qpt1*∆). Thus, *qpt1* deletion can partially restore the DNA damage sensitivity of *hst3*∆ *hst4*∆ cells in a Tdh2-dependent manner. Next, we examined whether *qpt1* deletion was able to extend the lifespan of *sir2*∆ cells by pedigree analysis. As shown in Fig. 3d, *sir2*∆ *qpt1*∆ cells experienced significantly extended replicative lifespans compared to *sir2*∆ cells (*sir2*∆ *qpt1*∆ *vs*. *sir2*∆ (*P* = 8.324E-65)). Furthermore, the lifespan of *sir2*∆ *tdh2*∆ *qpt1*∆ cells was almost the same as that of *sir2*∆ *tdh2*∆ cells (Fig. 3e; *sir2*∆ *tdh2*∆ *qpt1*∆ vs. *sir2*∆ *tdh2*∆), suggesting that *qpt1* deletion extends the lifespan of *sir2*∆ in a Tdh2-dependent manner. Next, we investigated whether the *qpt1* deletion suppressed spontaneous *URA3* gene deletion between DRs as well as the *tdh2* deletion. The ratio of *CaURA3* gene deletion in DRs was significantly (*P* = 0.042) reduced in *qpt1*∆ cells compared to wild-type cells (Fig. 3f). Altogether, these findings suggest that the elevated intracellular QUIN levels in the *tdh2* deletion contribute to genome stability.

### Intracellular NAD^+^, which is elevated in ***tdh2***∆ cells, does not suppress the DNA damage sensitivity of ***hst3***∆ ***hst4***∆ cells

QUIN is utilized in the NAD^+^ salvage pathway to synthesize NAD^+^ (Fig. S5A). We hypothesized that QUIN accumulated to synthesize NAD^+^ de novo and that NAD^+^ functioned as an end product to suppress replication fork instability in *tdh2*∆ cells. To test this hypothesis, we examined whether *tdh2* deletion elevated intracellular NAD^+^ levels. The cells were cultured, and the intracellular NAD^+^ concentration was measured by performing acid extractions of nucleotides over the course of cell growth in liquid cultures. The intracellular NAD^+^ concentration was almost the same between wild-type and *tdh2*∆ cells at first, although the concentration was severely decreased in cells lacking *NPT1*, the nicotinate phosphoribosyl transferase in the NAD^+^ salvage pathway (Fig. S5A and B)^41^. Intracellular NAD^+^ concentrations in both wild-type and *npt1*∆ cells steadily declined over the course of cell culture (Fig. S5B) because yeast cultures reached the end of the log phase and approached the diauxic shift, which was due to depletion of the limiting NAD^+^ precursor nicotinic acid from the growth medium^41,42^. In contrast, the NAD^+^ concentration remained constant in *tdh2*∆ cells over the course of cell culture (Fig. S5B), indicating that *tdh2* deletion promotes NAD^+^ synthesis. In addition, *tdh2* deletion slightly increased the intracellular NAD^+^ concentration in *hst3*∆ *hst4*∆ *tdh2*∆ cells, although the concentration declined in *hst3*∆ *hst4*∆ cells (Fig. S5C). Next, we examined whether de novo NAD^+^ synthesis induced by *tdh2* deletion is necessary for the growth of *hst3*∆ *hst4*∆ cells. To monitor the contribution of *tdh2* deletion to declining intracellular NAD^+^ levels in *hst3*∆ *hst4*∆ cells, we constructed *npt1*∆ *hst3*∆ *hst4*∆ and *npt1*∆ *hst3*∆ *hst4*∆ *tdh2*∆ strains containing the PHM286 plasmid harboring the *HST3* and *URA3* genes. Strains were streaked on SC solid medium supplemented with 5-FOA, a counterselecting agent, to induce loss of the *URA3* plasmid. Although *npt1*∆ *hst3*∆ *hst4*∆ cells did not grow in SC medium with 5-FOA, *npt1*∆ *hst3*∆ *hst4*∆ *tdh2*∆ cells did grow (Fig. S5D). Thus, an increase in intracellular NAD^+^ levels by *tdh2* deletion is necessary for the cell growth of *hst3*∆ *hst4*∆ cells. To reveal the mechanism that elevates intracellular NAD^+^ levels by *tdh2* deletion, we compared the transcription levels of genes involved in NAD^+^ synthesis among strains. The *BNA2* and *BNA4* genes belong to the kynurenine pathway and provide QUIN for tryptophan (Fig. 3a), and *TNA1* encodes the high affinity nicotinic acid plasma membrane permease responsible for the uptake of nicotinic acid (Fig. S5A). Although the expression levels of the *BNA2* and *BNA4* genes were almost the same between wild-type and *tdh2*∆ cells and the level of *TNA1* was slightly increased in *tdh2*∆ cells compared with wild-type cells (Fig. S5E), the expression levels of these genes were significantly (*P* < 0.05) increased in *hst3*∆ *hst4*∆ *tdh2*∆ cells compared with *hst3*∆ *hst4*∆ cells (Fig. S5E and F). Thus, *tdh2* deletion induces the gene expression involved in intracellular NAD^+^ synthesis in *hst3*∆ *hst4*∆ cells, which can improve the cell growth of *hst3*∆ *hst4*∆ cells.

Next, we examined whether elevated intracellular NAD^+^ levels suppressed the DNA damage sensitivity of *hst3*∆ *hst4*∆ cells. Isonicotinamide (INAM), an isostere of nicotinamide, raises intracellular NAD^+^ levels in budding yeast^43^. As shown in Fig. S6A, the intracellular NAD^+^ concentration was significantly (*P* < 0.05) elevated in the presence of INAM in both wild-type and *hst3*∆ *hst4*∆ cells. Treatment with INAM allowed both wild-type and *hst3*∆ *hst4*∆ cells to tolerate MMS (Fig. S6B). However, the addition of INAM did not suppress the sensitivities of *hst3*∆ *hst4*∆ cells to HU and CPT (Fig. S6B). These results indicate that elevated intracellular NAD^+^ levels do not contribute to the DNA damage sensitivity of *hst3*∆ *hst4*∆ cells. Altogether, these data suggest that NAD^+^ does not contribute to the suppression of replication fork instability in *tdh2*∆ cells.

## Discussion

In this study, we elucidated that the deletion of *TDH2* suppresses replication fork instability in the chromatin environment in which composed histones were acetylated. The acetylation of histone molecules loosens the chromatin structure by weakening the DNA-histone interaction. In particular, histone H3 on K56 is located within the histone core region and interacts with the DNA strand, and acetylation directly weakens the DNA-histone interaction^16^. The loosened chromatin structure becomes fragile, causing SSBs and DSBs during nuclear activities and exposes the naked DNA region to possible replication fork slippage at repeats. Both *sir2*∆ and *hst3*∆ *hst4*∆ cells exhibit high frequencies of loss of heterozygosity (LOH)^20,44^. LOH occurs in diploid cells that lose the chromosome arm or regenerate another chromosome arm instead of losing the arm by break-induced replication (BIR)^4,45^. In this study, *tdh2* deletion suppresses recombination by replication fork slippage in both *hst3*∆ *hst4*∆ and *sir2*∆ cells. The frequency of recombination between DRs was reduced in *tdh2*∆ cells compared to wild-type cells (Fig. 2c), which contributes to the extension of the replicative lifespan of *tdh2*∆ cells (Fig. 2a)^26^.

ROS, hazardous byproducts of mitochondrial respiration, are well recognized as mediators of DNA damage^46^. DNA damage caused by ROS appears as an oxidized base, a sugar modification, a DNA or protein crosslink or a DNA strand break^47–49^. 8-Oxoguanine, a representative oxidized DNA adduct, works as a replication fork blocker^50,51^. The accumulation of DNA damage causes replication fork stalling, which provides an opportunity for HR between direct repeats on the DNA strand. DNA damage agents used in this study (MMS, HU and CPT) are involved in ROS production. MMS indirectly inhibits respiratory chain in mitochondria by caused mitochondrial DNA (mtDNA) damage, which induces ROS production in budding yeast^52^. CPT also inhibits DNA topoisomerase I in mitochondria to induce mtDNA damages, which inhibits the respiratory chain in mitochondrion to induce ROS production in mammals^53^. HU also induces ROS production in budding yeast^54^. ROS is usually scavenged by several cellular metabolites, NADPH, a reduced form of nicotinamide adenine dinucleotide phosphate (NADP), functions as a major scavenger of ROS and is provided from the pentose phosphate pathway (PPP) branched from glycolysis mainly. In addition of generating phosphopentoses and ribonucleotides, PPP plays a pivotal role to combat oxidative stress^55^. Our previous study showed that *tdh2*∆ and *hst3*∆ *hst4*∆ *tdh2*∆ cells accumulated NADP^+^, metabolic intermediates of PPP and various ribonucleotides^26^. The phenotype of *tdh2*∆ is similar to that of *fbp1*∆, essential gene in gluconeogenesis^26^. The combination with interruption of gluconeogenesis caused by *fbp1*∆ and triple sirtuin gene deletions (*hst3*∆ *hst4*∆ *sir2*∆) alters the metabolic flux from glycolysis to PPP and increase the ribonucleotide levels^56^. These suggests that *tdh2*∆ alters the metabolic flux from glycolysis to PPP, and increases NADPH level to scavenge ROS. Therefore, *tdh2*∆ brings the resistance for DNA damage agents (HU, MMS and CPT) in *hst3*∆ *hst4*∆ genetical background, and stabilizes replication fork to prevent homologous recombination between DRs.

In mammalian, QUIN has been shown to increase the production of free radicals, leading to oxidative stress, DNA damage, and increased poly(ADP-ribose) polymerase 1 (PARP-1) activity^57,58^ . However, moderate QUIN level can induce resistance to oxidative stress through increased NAD^+^ production^59^. NAD^+^ influences DNA repair and gene expression through its role as a substrate for PARP-1 in mammal cells. Although there is no PARP in budding yeast, another mechanism supports to allow QUIN to induce resistance for oxidative stress. It has been shown that QUIN chelates ferrous iron to generate ROS^60^. Iron–Sulfur (Fe–S) centers are metallic cofactors with electronic properties that are associated with proteins^61^. It is well known that numerous DNA-transacting proteins such as DNA replication machinery contains Fe–S centers^54^. Because Fe–S centers are sensitive to oxidative agents, a moderate QUIN level can activate the oxidative stress response, and leads to resistance for oxidative stress. In the future, approaches to repair or prevent DNA damage will focus more heavily on altering the metabolic state of cells.

## Methods

### Strains and media

The genotypes of the strains, plasmids and primers used in this study are listed in Table S1. The parental budding yeast strain used in the present study was BY4742 (*MATα his3∆ leu2*∆*1 met15*∆*0 ura3*∆*0*)^62^. Yeast cells were routinely grown at 30 °C in YPD (1% yeast extract, 2% peptone, 2% glucose) or appropriate synthetic complete (SC) medium^63,64^. If necessary, the media were solidified with 2% agar. A yeast strain harboring a single gene deletion was commercially available from the haploid yeast open reading frame deletion collection^65^ (GE Dharmacon, Lafayette, CO, USA). To construct a double or triple gene deletion strain, the different mating type single gene deletion haploid strains were crossed, and sporulation was subsequently induced. After dissection, the spores were germinated on YPD medium. The deletion of each gene was confirmed using either antibiotics or auxotrophic markers and checking for growth on agar plates containing antibiotics or SC agar plates without selective amino acids. We employed YPD media supplemented with the following antibiotics: G418 (Sigma-Aldrich, St. Louis, MO, USA) at a final concentration of 100 μg/ml for the *kan* gene, hygromycin B at a final concentration of 200 μg/ml for the *hph* gene and ClonNAT (Werner Bioagents, Germany) at a final concentration of 100 μg/ml for the *nat* gene. SC-histidine medium was employed to select the *his5*^+^ strain^66,67^. The strains with deletions in both *HST3* and *HST4* harbor the PHM286 *URA3* plasmid, which contains the wild-type *HST3* gene and prevents spontaneous DNA damage and genomic instability. These strains were counterselected for loss of the PHM286 plasmid by selecting colonies that grew in SC medium supplemented with 5-fluoroorotic acid (5-FOA) at a final concentration of 100 µg/ml prior to use in subsequent experiments. To construct the strains monitoring *CaURA3* gene deletion frequencies, the plasmid PHM764 was digested with *Bsu* 36I and integrated into the *TRP1* locus. The URA + strains were selected and confirmed for correct integration of the PHM764 plasmid by PCR.

A standard method was used for isolation of the yeast genomic DNA^64^. *E. coli* strain DH5α^68^ and standard media and methods were used for plasmid manipulations^69^. Plasmid DNA was isolated from *E. coli* using a QIAquick Spin Miniprep kit (Qiagen, Santa Clarita, CA, USA). DNA fragments from polymerase chain reaction (PCR) samples or agarose gels were isolated using the Wizard SV Gel and PCR Clean-up kit (Promega, Madison, WI, USA). Oligonucleotides were purchased from either Invitrogen (Invitrogen, Carlsbad, CA, USA) or FASMAC (FASMAC, Kanagawa, Japan).

### Plasmid construction

DNA for plasmid construction was generated by PCR using the iProof High-Fidelity DNA polymerase (Bio-Rad, Hercules, CA, USA). The mix contained 10 µl of 5 × iProof buffer, 0.25 µl each of 100 µM PCR primer, 1 μl of 10 mM dNTP mix, 0.1 µg of template DNA and 0.5 µl of iProof Taq polymerase (final volume 50 μl). Reactions were run for 1 cycle of 10 s at 98 °C, 25 cycles of 10 s at 98 °C, 10 s at 55 °C, and 1 min/kb of desired product at 72 °C. These 25 cycles were followed by a 5-min extension at 72 °C. To construct plasmid PHM764, an ~ 2400 bp DNA fragment (*Candida albicans URA3* gene flanking region with each 500 bp direct repeat) in pAG61^70^ was digested with *Bam* HI and *EcoR* V and then ligated into *Bam* HI/*Hinc* II-digested YIplac204 plasmid^71^.

### DNA damage sensitivity test

Yeast strains were cultured in 5 ml of YPD liquid medium at 25 °C overnight and then adjusted to 5 × 10^6^ cells/ml in 5 ml of YPD medium. Next, cell culture was continued for 3 h at 25 °C, and then cells were harvested and suspended in DIW at 5 × 10^7^ cells/ml. Two hundred microliters of cell suspension (1 × 10^7^ cells) was transferred to a 96-well plate and sequentially diluted tenfold to a concentration of 5 × 10^3^ cells/ml. A small portion of the diluted cell suspension in each well was set on YPD medium containing each concentration of DNA damaging agent using a replica plater (Sigma-Aldrich, St. Louis, MO, USA). The plates were incubated at 25 °C for 3 to 5 days. YPD media was supplemented with the following DNA damaging agents: methyl methanesulfonate (MMS), hydroxyurea (HU) and camptothecin (CPT). The concentration of each agent is listed in figures.

### Western blotting

Proteins were separated by 15% sodium dodecyl sulfate–polyacrylamide gel electrophoresis (SDS-PAGE) and transferred to nitrocellulose membranes (Amersham PROTORAN) (GE healthcare, Little Chalfont, Buckinghamshire, England). The protein level in each lane on a nitrocellulose membrane was adjusted equally and confirmed by staining with 0.1% Ponceau S solution (Sigma-Aldrich, St. Louis, MO, USA). Rabbit polyclonal anti-acetylated lysine 56 on the yeast histone H3 antibody (1:1000 dilution) was used^16^. For detection of phosphorylated species of the Rad53 protein, anti-Rad53 rabbit polyclonal antibody (the equivalent antibody is commercially available (Santa Cruz Biotechnology, Dallas, TX, USA)) was used.

### Detection of Rad53 phosphorylation in response to DNA damage

Yeast strains were cultured in 5 ml of YPD liquid medium at 25 °C overnight and then adjusted to a concentration of 5 × 10^6^ cells/ml in 5 ml of YPD medium containing MMS at a final concentration of 0.03%. Afterwards, cell culture was continued for 3 h at 25 °C, and then cells were harvested. The whole cell extraction method was described previously^72^. Multiple species of phosphorylated Rad53 and unphosphorylated Rad53 were separated by 7.5% SDS-PAGE and then detected by western blotting using an anti-Rad53 antibody.

### Cell cycle arrest during G2/M phase

Yeast strains were cultured in 5 ml of YPD liquid medium at 25 °C overnight and then adjusted to a concentration of 5 × 10^6^ cells/ml in 5 ml of YPD medium containing nocodazole at a final concentration of 10 µg/ml. Afterwards, cell culture was continued for 3 h at 25 °C, and then cells were harvested. The whole cell extraction method was previously described^72^.

### Replicative lifespan assay (pedigree analysis)

The pedigree analysis procedure was described previously^26^. Typically, a minimum of 50 mother cells was counted for each strain tested. To compare the difference in replicative lifespans among strains statistically, we performed the unpaired t-test (two-tailed).

### *CaURA3* deletion assay

The yeast strains were streaked on SC-Ura plates to select *URA3*-positive strains prior to the assay. The *URA3*-positive colonies were inoculated in 5 ml of YPD liquid medium and cultured at 25 °C overnight. A small aliquot of culture (5 × 10^4^ or 5 × 10^5^ cells/strain) was plated in a YPD solid plate supplemented with 5-FOA at a final concentration of 100 µg/ml to select strains containing the *ura3* gene deletion. The number of colonies was counted, and the frequencies of the *CaURA3* gene deletion were calculated for plated cells. Three replicates were analyzed for each strain.

### Synthetic lethality test

Yeast strains with or without the PHM286 plasmid (*URA3*) were grown on YPD medium at 25 °C overnight; then, the strains were streaked on SC solid medium supplemented with 5-FOA at a final concentration of 100 µg/ml at 25 °C for 3 days to counterselect for loss of the PHM286 plasmid.

### RNA isolation and real time (RT)–PCR

Total RNA was isolated from budding yeast using the RNeasy Mini Kit (Qiagen, Santa Clarita, CA, USA). A relative comparison of the mRNA amount was performed using a One Step SYBR PrimeScript PLUS RT-PCR Kit (Takara-Bio, Kusatsu, Shiga, Japan). The mix contained 10 µl of 2 × One Step SYBR RT-PCR buffer 4, 1.2 µl of Takara Ex Taq HS Mix, 0.4 µl of PrimeScript PLUS RTase Mix, 0.8 µl of 10 µM PCR forward primer, 0.8 µl of 10 μM PCR reverse primer and 100 ng of total RNA (final volume 20 µl). Reactions were run for 1 cycle of 5 min at 42 °C, 1 cycle of 10 s at 95 °C, 40 cycles of 5 s at 95 °C, 1 cycle of 20 s at 55 °C, 1 cycle of 1 s at 95 °C, and 1 cycle of 15 s at 65 °C, followed by 1 s at 95 °C using either a Light Cycler 480 System II or Light Cycler Nano (Roche Life Science, Penzberg, Germany). The level of each mRNA was compared with the amount of *ACT1* mRNA. PCR primers are listed in Table S1.

### The measurement of NAD^+^ concentration and INAM treatment

The yeast strains were cultured in 250 ml of YPD liquid medium at 25 °C. During cell culture, the OD_600 nm_ of a 1:10 cell dilution and the cell number were recorded, and 20 ml of culture was pelleted and washed with water. After harvesting, the cell pellet was stocked at − 80 °C until use. The preparation and quantification of intracellular NAD^+^ was described previously^41^. Over 10 independent cultures were routinely used to determine the NAD^+^ concentrations in duplicate.

For INAM treatment, the yeast strains (5 × 10^6^ cells/ml at start) were cultured in 20 ml of YPD liquid medium supplemented with or without INAM at a final concentration of 25 mM at 25 °C until an OD_600_ of ~ 1.5 was reached and then harvested as described previously^43^. Cell pellets were employed to measure the intracellular NAD^+^ concentration.

### Measurement of intracellular quinolinic acid (QUIN) concentration

For metabolite extraction, yeast cells (1 × 10^8^ cells) were suspended in 50% methanol and immediately frozen in liquid nitrogen. Then, frozen samples were ground by a Multi Beads Shocker (Yasui Kikai, Osaka, Japan) then centrifuged at 13,000 × g for 10 min at 4 °C. The supernatant was mixed with an equal volume of chloroform, and the mixture was centrifuged again. The upper aqueous phase was transferred to a tube and evaporated using SpeedVac SPD 1010 (Thermo Fischer Scientific, Waltham, MA, USA). Levels of quinolinic acid (QUIN) in yeast cells were determined using an Agilent 6460 Triple Quad mass spectrometer coupled to an Agilent 1290 HPLC system with multiple reaction monitoring (MRM) mode. The MRM transition for QA was optimized as m/z 166 to 78. MS settings and chromatographic conditions were described previously^73^. The amount of QA was calculated by integrating the sum of the area using Mass Hunter Quantitative software (Agilent Technologies, Santa Clara, CA, USA).

## Supplementary information

Supplementary Tables.

Supplementary Figures.
