# Supplementary material for: Deletion of the GAPDH gene contributes to genome stability in Saccharomyces cerevisiae
Source: Sci Rep. 2020 Dec 3;10:21146. doi: 10.1038/s41598-020-78302-5 (PMC7713361; doi:10.1038/s41598-020-78302-5)
Supplement: Supplementary file 2 — Supplementary Figures. [file 41598_2020_78302_MOESM2_ESM.docx]

Title: Deletion of the *GAPDH* gene contributes to genome stability in *Saccharomyces cerevisiae*

**Authors:** Miki Hanasaki^1^, Keisuke Yaku^2^, Motohiro Yamauchi^3^, Takashi Nakagawa^2^ and Hiroshi Masumoto^1^*

**Affiliations:**

^1^Biomedical Research Support Center (BRSC), Nagasaki University School of Medicine, 1-12-4 Sakamoto, Nagasaki, Nagasaki, 852-8523, Japan

^2^Department of Metabolism and Nutrition, Graduate school of Medicine and Pharmaceutical Sciences for Research, University of Toyama, 2630 Sugitani, Toyama, Toyama, 930-0194, Japan

^~~3~~^Department of Radiation Biology and Protection, Atomic Bomb Disease Institute, Nagasaki University. 1-12-4 Sakamoto, Nagasaki, Nagasaki, 852-8523, Japan

*Correspondence to: [himasumo@nagasaki-u.ac.jp](mailto:himasumo@sakura.cc.tsukuba.ac.jp)

**Supplementary data**

**
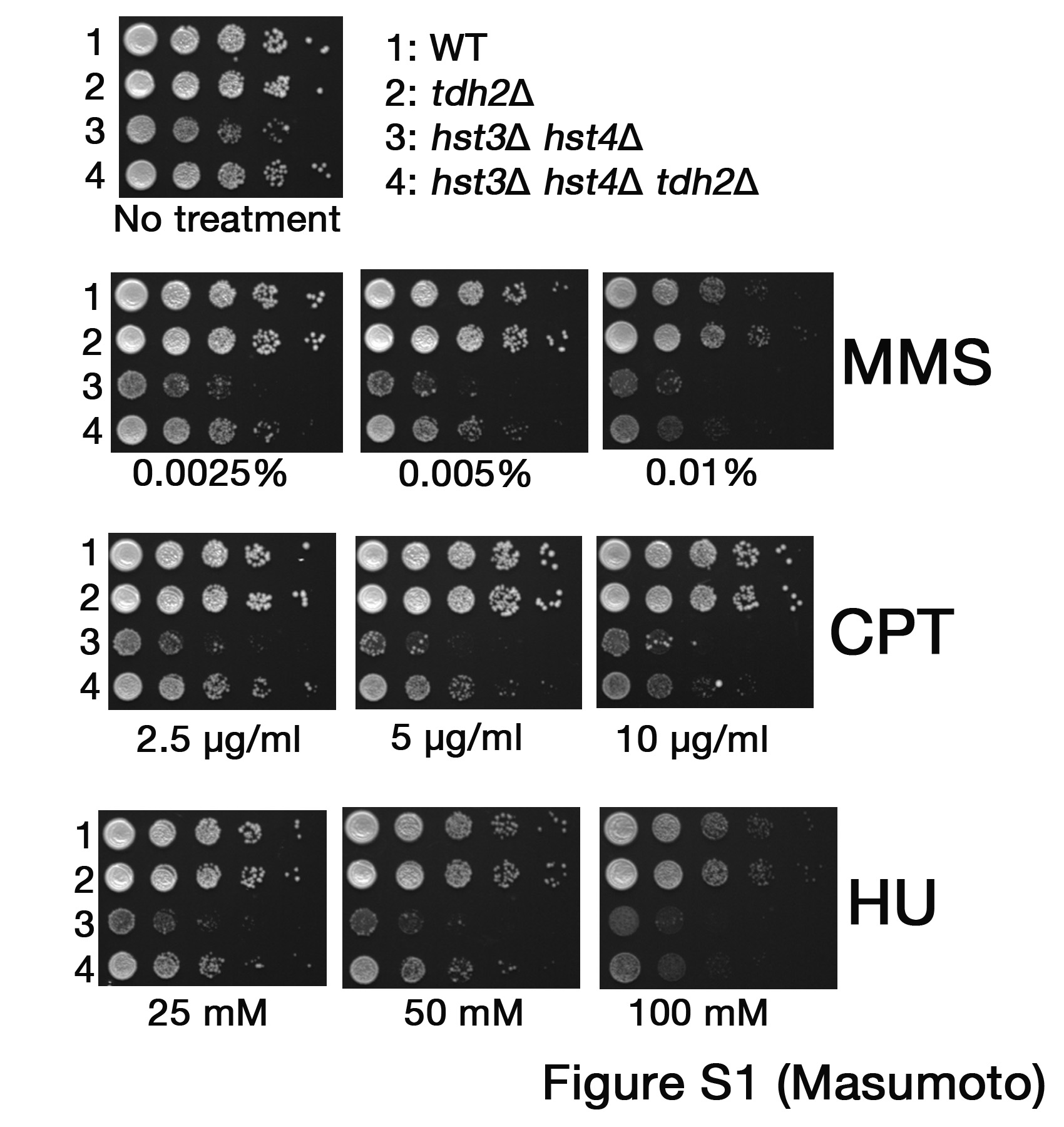
**

**Fig. S1.** Plate assays monitoring the sensitivity to each DNA damaging agents at each concentration are shown in Fig. 1a.

**
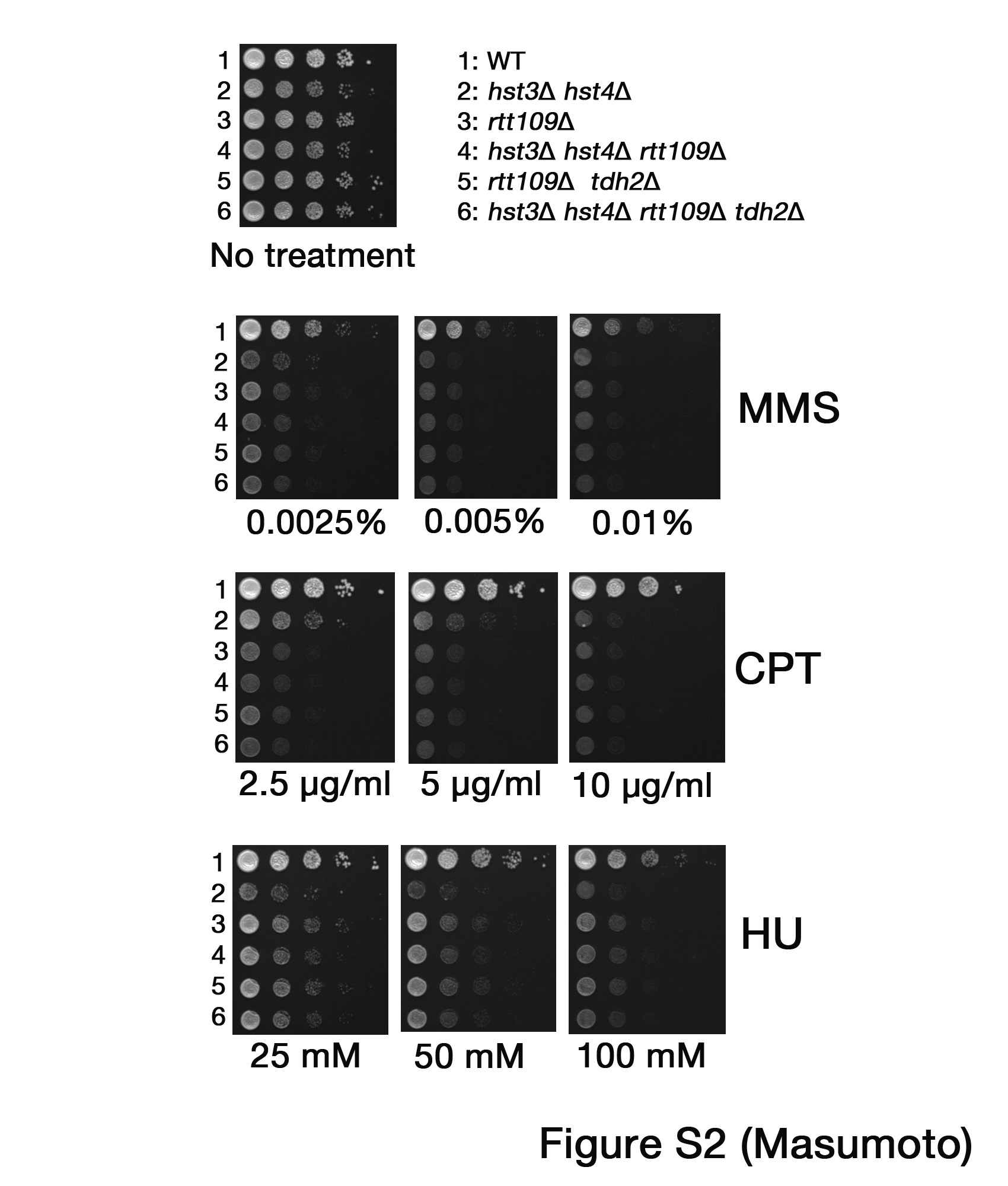
**

**Fig. S2.** Plate assays monitoring the sensitivity to each DNA damaging agent at each concentration are shown in Fig. 1c.

**
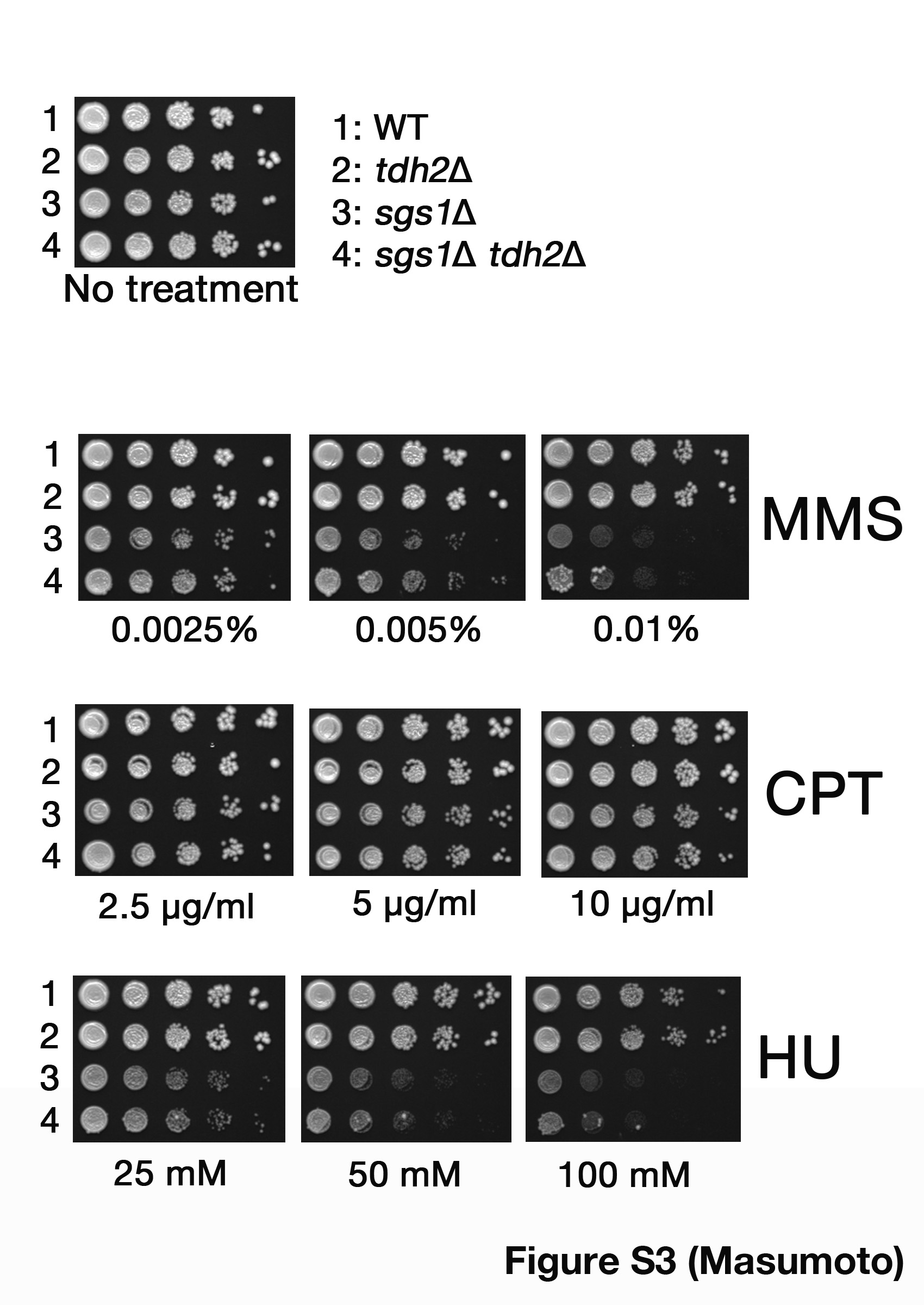
**

**Fig. S3.** Plate assays monitoring the sensitivity to each DNA damaging agent at each concentration.

**
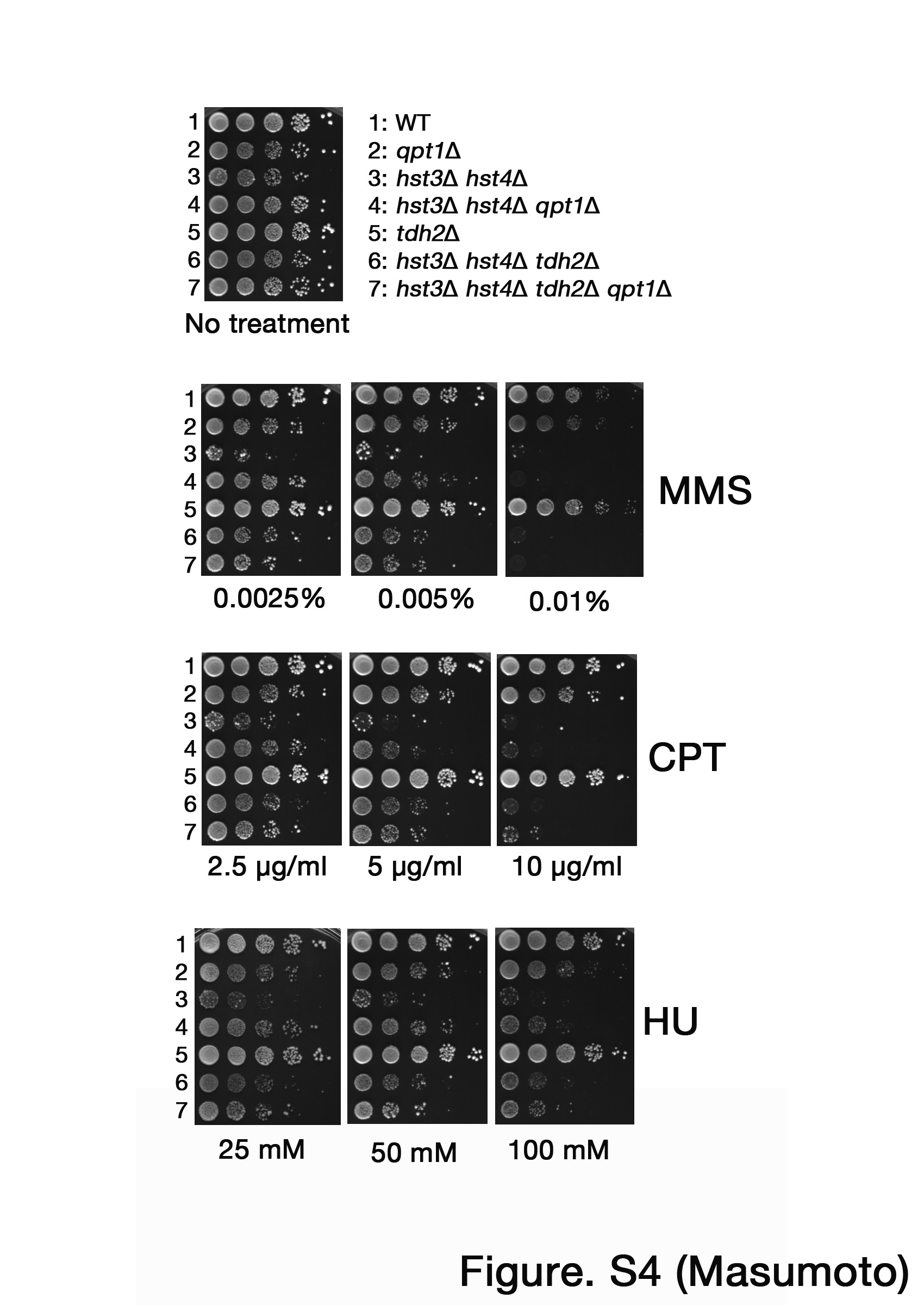
**

**Fig. S4.** Plate assays monitoring the sensitivity to each DNA damaging agent at each concentration are shown in Fig. 3c.

**
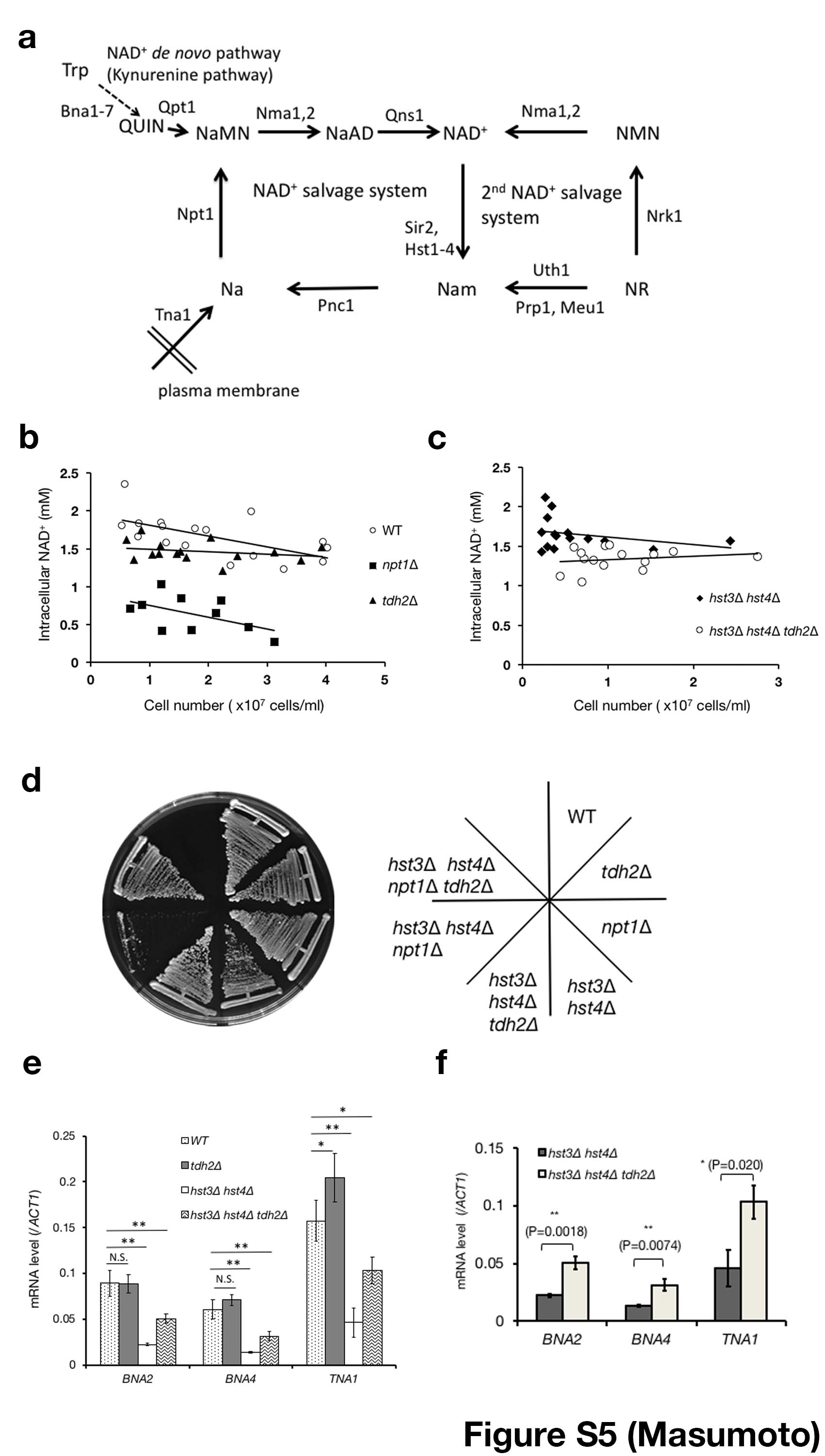
**

**Fig. S5. *tdh2* gene deletion elevates intracellular NAD^+^ concentration.** (a) The metabolic pathway of NAD^+^ synthesis (Belenky P*, et al.* ^1^). Trp: tryptophan. NaAD: nicotinic acid adenine dinucleotide. NMN: nicotinamide mononucleotide. NR: nicotinamide riboside. Nam: nicotinamide. Na: nicotinic acid. (b and c) Distribution of intracellular NAD^+^ concentration during cell growth. (d) Cell growth on SC+FOA plates. Cells were grown on solid YPD plates and streaked on SC medium supplemented with 5-FOA. (e and f) Comparison of mRNA levels among strains. (f) mRNA levels of both *hst3*∆ *hst4*∆ and *hst3*∆ *hst4*∆ *tdh2*∆ data were extracted from (e). **P*<0.05. ***P*<0.01. Repeated measures ANOVA with Bonferroni correction (e). Unpaired t-test (two-tails) (f). Error bars represent the standard deviation of three biological replicates.

**
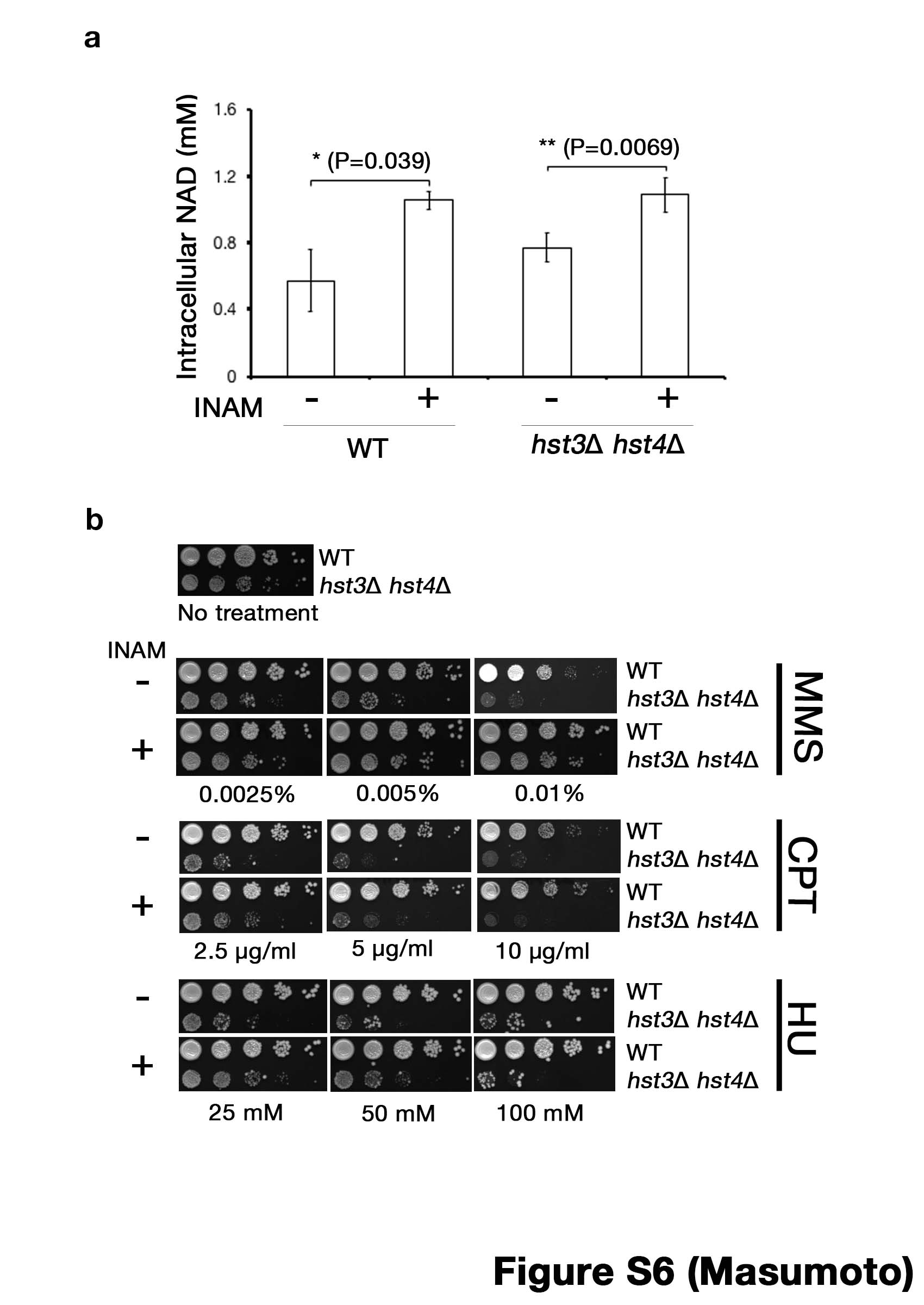
**

**Fig. S6. An increase in intracellular NAD^+^ cannot suppress the DNA damage sensitivity of *hst3*∆ *hst4*∆ cells.** (a) Isonicotinamide (INAM) elevates the intracellular NAD^+^ concentration in both wild-type and *hst3*∆ *hst4*∆ cells. **P*<0.05. ***P*<0.01. Unpaired t-test (two-tails). Error bars represent the standard deviation of four biological replicates. (b) Plate assays monitoring the sensitivity of each concentration of DNA damaging agent among cells with or without INAM.

**
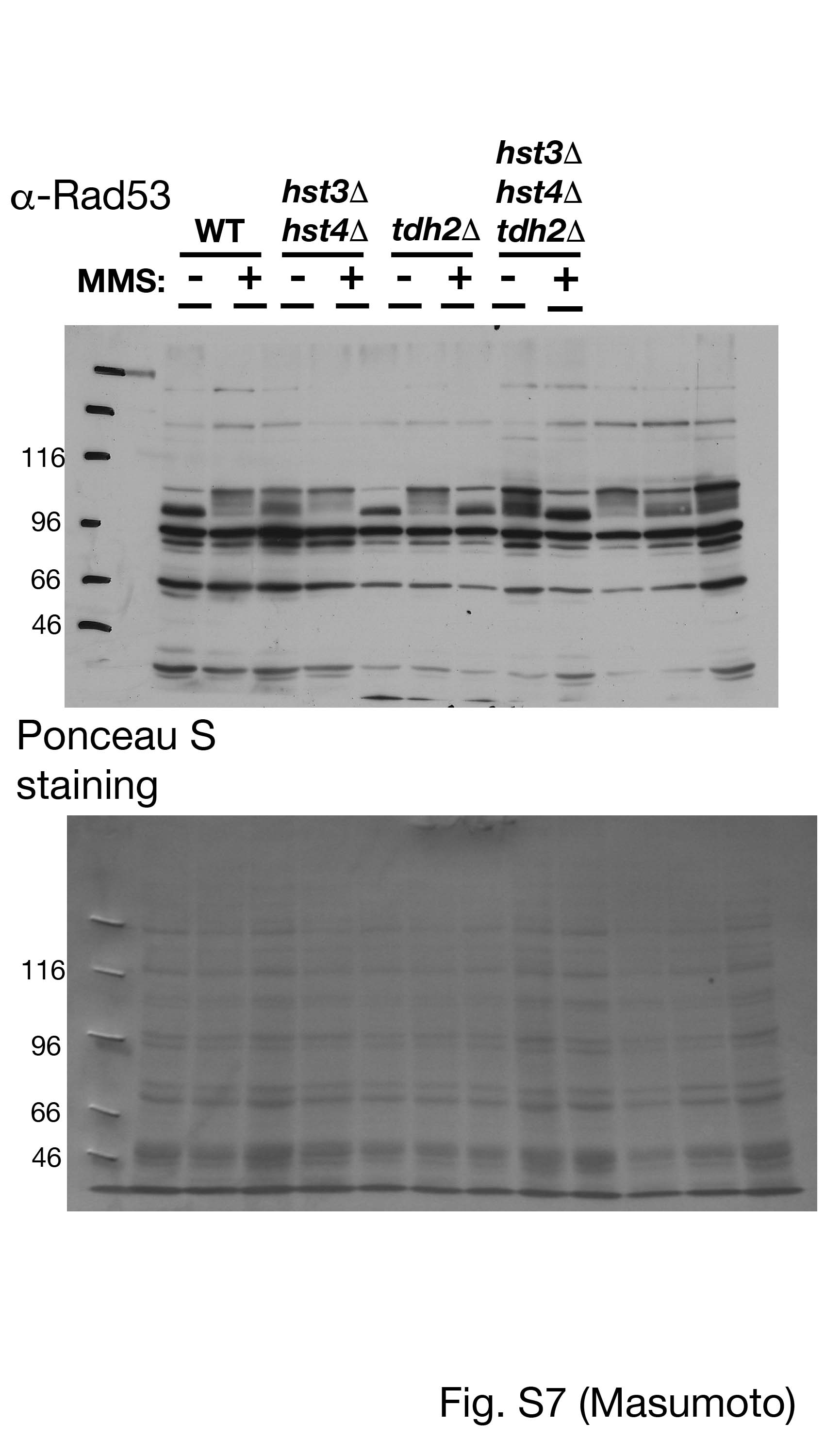
**

**Fig. S7. Full-length blots of Fig. 1b.**

**
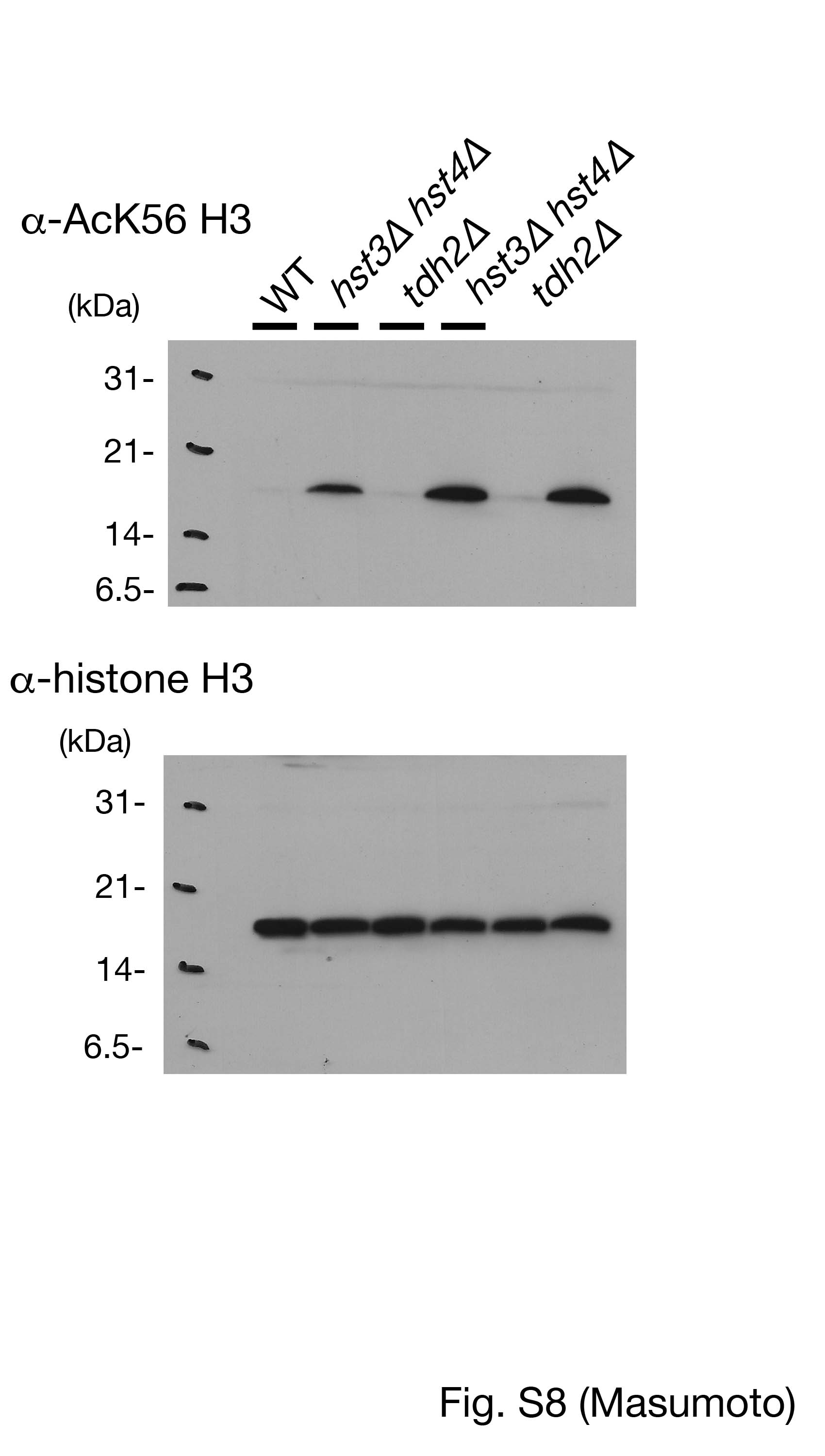
**

**Fig. S8. Full-length blots of Fig. 1d.**

**Table S1. Yeast strains, plasmids, primers, sequences.**

**Supplementary reference**

1 Belenky, P. *et al.* Nicotinamide riboside promotes Sir2 silencing and extends lifespan via Nrk and Urh1/Pnp1/Meu1 pathways to NAD+. *Cell* **129**, 473-484, doi:10.1016/j.cell.2007.03.024 (2007).
